# Association between Self-Reported Chewing Status and Glycemic Control in Japanese Adults

**DOI:** 10.3390/ijerph18189548

**Published:** 2021-09-10

**Authors:** Komei Iwai, Tetsuji Azuma, Takatoshi Yonenaga, Daisuke Ekuni, Kazutoshi Watanabe, Akihiro Obora, Fumiko Deguchi, Takao Kojima, Manabu Morita, Takaaki Tomofuji

**Affiliations:** 1 Department of Community Oral Health, School of Dentistry, Asahi University, 1851 Hozumi, Mizuho, Gifu 501-0296, Japan; ko-mei@dent.asahi-u.ac.jp (K.I.); tetsuji@dent.asahi-u.ac.jp (T.A.); yone0730@dent.asahi-u.ac.jp (T.Y.); 2 Department of Preventive Dentistry, Graduate School of Medicine, Dentistry and Pharmaceutical Sciences, Okayama University, 2-5-1 Shikata-cho, Kita-ku, Okayama 700-8558, Japan; dekuni7@md.okayama-u.ac.jp (D.E.); mmorita@md.okayama-u.ac.jp (M.M.); 3 Medical Check-Up Center, Asahi University Hospital, 3-23 Hashimoto-cho, Gifu 500-8523, Japan; watanabe@hosp.asahi-u.ac.jp (K.W.); akiobora18@hosp.asahi-u.ac.jp (A.O.); deguchi5757@yahoo.co.jp (F.D.); tkojima-gi@umin.ac.jp (T.K.)

**Keywords:** mastication, glycated hemoglobin A, epidemiology, cross-sectional studies

## Abstract

This cross-sectional study investigated the relationship between self-reported chewing status and glycemic control in 30,938 Japanese adults who participated in health checkups. Chewing status was evaluated using a self-reported questionnaire. We defined high hemoglobin A1c (HbA1c) levels as a HbA1c level ≥6.5%; 692 (2.2%) respondents met this criterion. After adjusting for gender, age, smoking status, exercise habits, body mass index and eating speed, high HbA1c levels was found to be associated with male gender (odds ratio (OR), 1.568; 95% confidence interval (CI), 1.310 to 1.878; *p* < 0.001), older age (OR, 1.077; 95% CI, 1.068 to 1.087; *p* < 0.001), higher body mass index (OR, 1.246; 95% CI, 1.225 to 1.268; *p* < 0.001), current smoker status (OR, 1.566; 95% CI, 1.303 to 1.882; *p* < 0.001) and chewing difficulty (OR, 1.302; 95% CI, 1.065 to 1.591; *p* < 0.05). In conclusion, self-reported chewing difficulty was associated with high HbA1c levels in Japanese adults.

## 1. Introduction

Diabetes is a lifestyle-related disease characterized by high blood sugar levels and it leads to complications, such as neuropathy, retinopathy and nephropathy [1]. The prevalence of diabetes continues to increase worldwide; in 2017, approximately 451 million people worldwide had diabetes and the number is expected to reach 693 million by 2045 [2]. According to the 2019 National Health and Nutrition Survey [3] in Japan, about 20% of men and 11% of women have or are likely to have diabetes and the public health implications are enormous.

Hemoglobin A1c (HbA1c) serves as a biomarker for testing and monitoring diabetes, because it reflects changes in blood glucose levels [4] and it can be applied to guide strategies for diabetes treatment and to control and predict the risk of progressive complications of diabetes [5]. Hence, the monitoring of HbA1c is important for the development of appropriate strategies to prevent diabetes and its associated complications.

Some studies have reported relationships between chewing status and glycemic control. For instance, a clinical study reported that insufficient chewing status was more frequently seen in subjects with a HbA1c level ≥7% than in those with a level <7% [6]. It is also known that difficulties in chewing status may result in lower intakes for key nutrients as iron and fiber, contributing to induce poor glycemic control [7]. These reports are judgments based on a small number of limited number of subjects who used special devices such as a Gluco Sensor to evaluate their masticatory status. On the other hand, since it is time-consuming to measure the chewing status of each individual using devices, screening the chewing status using a questionnaire is common in a health checkup for a large group of people. Therefore, it is also important to evaluate the relationship between self-reported chewing status and glycemic control in humans. However, it is unclear the relationship between self-reported chewing status and the glycemic status.

In Japan, the Ministry of Health, Labor and Welfare has mandated that medical insurers must require insured persons 40 to 74 years of age to undergo specific health checkups focused on metabolic diseases, including diabetes mellitus. Unfortunately, the measurements of chewing status using the devices are not conducted in specific health checkups. However, among the questionnaires used in the specific health checkups, an item on chewing status was added in 2018. Investigating the relationship between self-reported chewing status and glycemic control is important in considering the necessity of dental intervention in health guidance after specific health checkups. The present study examined whether the self-reported chewing status is associated with glycemic control in a Japanese population that underwent regular health checkups [8].

## 2. Materials and Methods

### 2.1. Participants

We performed a multi-center cross-sectional study. We analyzed data from community residents who participated in health checkups at Asahi University Hospital in Gifu, Japan and at the Junpukai Health Maintenance Center in Okayama, Japan. A total of 35,571 Japanese adults aged ≥20 years participated in the baseline survey between April 2018 and March 2019. We excluded 521 residents due to missing data on the HbA1c level. We also excluded 2913 residents who did not complete questionnaires and 1199 residents who take glucose-lowering drugs. In total, we analyzed data from 32,137 community residents in this study. This study was approved by the Ethics Committee of Asahi University (No. 27010) and was performed in accordance with the Declaration of Helsinki. All residents who participated provided written informed consent. Study reporting conformed to STROBE guidelines.

### 2.2. Measurement of HbA1c

The measurement of HbA1c levels was done using high-performance liquid chromatography with venous blood samples [9].

### 2.3. Evaluation of Glycemic Control

In general, a HbA1c level ≥6.5% indicates high HbA1c levels [10]. Therefore, the participants who had a HbA1c level ≥6.5% were defined as having high HbA1c levels.

### 2.4. Assessment of Body Composition

The height and body weight were measured and the BMI was calculated as the weight (kg) divided by the height squared (m^2^).

### 2.5. Questionnaire

We used the same questionnaire that is used in medical checkups in Japan. The questionnaire items included age, gender, taking glucose-lowering drugs (absence/presence), current smoking status (yes/no), regular exercise habits (absence/presence), physical activity (low/high), adequate sleep (yes/no), alcohol consumption (not daily/daily), eating speed (slowly, medium, or quickly) and chewing status (“I can eat anything,” “Sometimes it is difficult to chew due to dental problems, such as dental caries and periodontal disease,” or “I can hardly chew.”) [11]. In this study, because the number of participants who answered “I can hardly chew” was small (*n* = 74), we combined the categories of “Sometimes it is difficult to chew due to dental problems, such as dental caries and periodontal disease” and “I can hardly chew”. Respondents who answered “Sometimes it is difficult to chew due to dental problems, such as dental caries and periodontal disease” or “I can hardly chew” were considered to have chewing difficulty [11].

### 2.6. Statistical Analysis

Continuous variables are expressed as medians (first and third quartiles). Significant differences in characteristics between the study participants with and without high HbA1c levels were assessed using the chi-squared and Mann-Whitney U tests. Univariate and multivariate stepwise logistic regression analyses were performed with the presence and absence of high HbA1c levels as dependent variables. The third category of variables concerned the sample (age, gender and BMI) and different variables related to glycemic control (smoking habits, exercise habits, drinking habits and eating speed) and these variables were adjusted for in these analyses. Since BMI was expected to have either an effect or little effect on the association between lifestyle and glycemic control, two analytical models were used, one with and one without BMI as an adjustment factor. Variables with a *p* < 0.10 were removed from the model while those with a *p* < 0.05 were included. Independent variables with a *p* < 0.05 in the univariate model were selected. All data were analyzed using SPSS statistics version 25 (IBM Japan, Tokyo, Japan). All *p* values <0.05 were considered to be statistically significant.

## 3. Results

Table 1 shows the numbers of participants with different HbA1c levels and chewing status. The numbers of participants with high HbA1c levels were 17 (0.5%) in the 20-to-39-year-old group, 545 (2.1%) in the 40-to-64-year-old group, 130 (5.9%) in the ≥65-year-old group and 692 (2.2%) in total among all participants. The numbers of participants who reported having chewing difficulty were 287 (9.3%) in the 20-to-39-year-old group, 3273 (12.8%) in the 40-to-64-year-old group, 406 (18.49.0%) in the ≥65-year-old group and 3966 (12.8%) in total among all participants.

The prevalence of high HbA1c levels was 2.2% (Table 2). Respondents with high HbA1c levels were significantly more likely to be male (*p* < 0.001), be older (*p* < 0.001), have a higher body mass index (BMI; *p* < 0.001), be a current smoker (*p* < 0.001), exercise regularly (*p* < 0.05), eat quickly (*p* <0.01) and have a chewing difficulty (*p* < 0.001).

Table 3 shows the results of univariate logistic regression analysis with high HbA1c levels as the dependent variable in all participants. The results showed that the odds ratio (OR) for high HbA1c levels was higher among those who were male (OR, 2.457; 95% confidence interval (CI), 2.076 to 2.908), were older (OR, 1.066; 95% CI, 1.058 to 1.075), had a higher BMI (OR, 1.235; 95% CI, 1.215 to 1.254), current smoker (OR, 1.802; 95% CI, 1.524 to 2.131), being absence of regular exercise habits (OR, 0.819; 95% CI, 0.691 to 0.970) and ate quickly (OR, 1.279; 95% CI, 1.098 to 1.489) than among those who did not. The results also showed that the OR for high HbA1c levels was higher among those who had chewing difficulty (OR,1.671; 95% CI, 1.381 to 2.023) than among those who did not.

Table 4 shows the results of the multivariate adjusted logistic regression analysis with high HbA1c levels as the dependent variable in all participants. High HbA1c levels was significantly associated with male gender (OR, 2.120; 95% CI, 1.777 to 2.529), older age (OR, 1.070; 95% CI, 1.061 to 1.079), current smoker status (OR, 1.607; 95% CI, 1.345 to 1.921), eating quickly (OR, 1.302; 95% CI, 1.115 to 1.520) and chewing difficulty (OR, 1.297; 95% CI, 1.067 to 1.576) after adjusting for gender, age, smoking status, regular exercise habits, eating speed and chewing status. After additional adjustments for the BMI, high HbA1c levels was significantly associated with male gender (OR, 1.568; 95% CI, 1.310 to 1.878), older age (OR, 1.077; 95% CI, 1.068 to 1.087), higher BMI (OR, 1.246; 95% CI, 1.225 to 1.268), current smoker status (OR, 1.566; 95% CI, 1.303 to 1.882) and chewing difficulty (OR, 1.302; 95% CI, 1.065 to 1.591).

## 4. Discussion

To the best of our knowledge, this is the first investigation of the association between the self-reported chewing status and glycemic control among Japanese adults. We found that self-reported chewing difficulty was associated with high HbA1c levels after adjusting for the potential confounding variables of gender, age, BMI, smoking status, regular exercise habits, eating speed and chewing status. The results indicated that maintaining good chewing status could contribute to better glycemic control. In Japan, there are no mandatory dental health checkups for adults. However, in the questionnaires used for the specific health checkups required for health insurance, there is an item on chewing status. This item on chewing status may be applied to screen for individuals who may need dental intervention for controlling blood sugar levels.

In this study, self-reported chewing difficulty was associated with high HbA1c levels. In terms of the relationship between self-reported questionnaires and glycemic control, a report from Qatar showed that diabetes was associated with those who had difficulty chewing by questionnaire [12]. These data support the concept that asking self-reported status is useful for evaluating glycemic and diabetic conditions.

There are several possible mechanisms for the relationship between the chewing status and glycemic control. Chewing breaks down big food particles into small pieces and this increases its digestibility by increasing the surface area for various enzymes to act on [13]. This helps to stimulate the cephalic phase of insulin secretion and incretin release from the gut to promote the absorption of glucose [13]. It is also known that chewing can attenuate decreases in the concentration of glucagon-like peptide-1 [14], which contributes to regulating glucose-stimulated insulin secretion [15]. As such, chewing is likely to regulate blood glucose levels through the secretion of insulin and incretin. However, because we did not measure the blood levels of insulin and incretin in this study, further studies are needed to clarify the mechanisms by which chewing status affects glycemic control.

In this study, high HbA1c levels was associated with aging and current smoking. These observations are consistent with previously reported findings demonstrating that aging [16] and smoking [17,18] are risk factors for diabetes. In addition, our results showed that a higher BMI was associated with high HbA1c levels. This observation is also in agreement with the findings of a previous study, which reported that an increased BMI above the normal weight levels was associated with an increased risk of being diagnosed with a complication resulting from diabetes [19].

We also found that after adjusting for gender, age, smoking status, regular exercise habits, eating speed and chewing status in all participants, high HbA1c levels was associated with eating quickly. However, this association disappeared after further adjustment for the BMI. This indicated that the BMI overwhelmed the effects of eating speed on glycemic control. Since it has been reported that eating speed [20] are closely correlated with the BMI, eating speed is thought to indirectly influence glycemic control by affecting the BMI.

In the present study, the prevalence of high HbA1c levels and chewing difficulty was 2.2% and 12.8%, respectively. From the data of the National Health and Nutrition Survey, the prevalence of high HbA1c levels and chewing difficulty was 14.5% and 25.0%, respectively, in 2019 [3]. This indicated that the prevalence of high HbA1c levels and chewing difficulty in our study were low when compared to the Japanese national data. The reason for these differences may be because our study was conducted among people who participated in medical checkups, suggesting that they may be more conscious of their health than those who do not participate in medical checkups. This may limit the extrapolation of our findings to the general population. In addition, we defined HbA1c level ≥6.5% as high HbA1c levels. In elderly and frail diabetic patients, it is recommended to set higher HbA1c thresholds than in young patients [21,22]. Therefore, separate HbA1c cutoffs for different age groups may be more appropriate [22].

The present study also has other limitations. For instance, the current study was a cross-sectional study, so it cannot demonstrate causal relationships. Additional longitudinal studies are needed to investigate the relationship between chewing status and glycemic control. In addition, since the specific health checkups did not include dental examination, we could not collect data about oral parameters including number of teeth present, presence of periodontal disease, actual chewing times and bite forces. Furthermore, HbA1c levels are not the preferred method for diagnosis of type II diabetes and that it is more of a monitoring test for patients with previously diagnosed diabetes. Since only HbA1c data was used as an indicator of glycemic control in our study, we cannot state the relationship between chewing status and the presence or absence of diabetes. On the other hand, a strength of this study is the sufficient sample size for assessing the prevalence of high HbA1c levels among participants with different chewing statuses. Furthermore, we were able to gather study population data from multiple locations (Gifu and Okayama).

## 5. Conclusions

In the present population, high HbA1c levels was associated with the self-reported chewing difficulty, male gender, older age, a higher BMI and current smoker status.

## Figures and Tables

**Table 1:** Numbers of participants with different HbA1c levels and chewing status.

Variables	20–39 Years (*n* = 3099)	40–64 Years (*n* = 25,635)	≥65 Years (*n* = 2204)	Total (*n* = 30,938)
HbA1c levels (%)				
<5.5	1855 (59.9)	10,389 (40.5)	330 (15.0)	12,574 (40.6)
5.5–5.9	1162 (37.5)	12,573 (49.0)	1233 (55.9)	14,967 (48.4)
6.0–6.4	66 (2.1)	2128 (8.3)	511 (23.2)	2705 (8.7)
6.5–6.9	10 (0.3)	311 (1.2)	98 (4.4)	419 (1.4)
7.0–7.4	3 (0.1)	84 (0.3)	19 (0.9)	106 (0.3)
7.5–7.9	2 (0.05)	44 (0.2)	6 (0.3)	52 (0.2)
≥8.0	2 (0.05)	106 (0.4)	7 (0.3)	115 (0.4)
<6.5	3082 (99.5)	25,090 (97.9)	2074 (94.1)	30,246 (97.8)
≥6.5	17 (0.5)	545 (2.1)	130 (5.9)	692 (2.2)
Chewing status (%)				
I can eat anything	2812 (90.7)	22,362 (87.2)	1798 (81.6)	26,972 (87.2)
Sometimes it is difficult to chew due to dental problems, such as dental caries and periodontal disease	280 (9.0)	3212 (12.5)	400 (18.1)	3892 (12.6)
I can hardly chew	7 (0.2)	61 (0.2)	6 (0.3)	74 (0.2)
Chewing difficulty	287 (9.3)	3273 (12.8)	406(18.4)	3966 (12.8)

**Table 2:** Characteristics of the study populations with and without high HbA1c levels.

Variables		High HbA1c Levels		*p* Value
		No (*n* = 30,246)	Yes (*n* = 692)	
Men, *n* (%) ^a^		15,671 (51.8)	502 (72.5)	<0.001
Age (years), medians (first and third quartiles) ^b^		49 (43, 56)	55 (49, 62)	<0.001
BMI (kg/m^2^), medians (first and third quartiles) ^b^		22.3 (20.3, 24.7)	26.1 (23.8, 29.1)	<0.001
Current smoker	Yes, *n* (%) ^a^	5471 (18.1)	197 (28.5)	<0.001
Regular exercise habits	Absence, *n* (%) ^a^	23,211 (76.7)	505 (73.0)	<0.05
Physical activity	Low, *n* (%) ^a^	22,283 (73.7)	516 (74.6)	0.629
Sleeping well	No, *n* (%) ^a^	11,300 (37.4)	271 (39.2)	0.341
Alcohol drinking	Everyday, *n* (%) ^a^	7604 (25.1)	173 (25.0)	0.962
Eating speed	Quickly, *n* (%) ^a^	11,281 (37.3)	299 (43.2)	<0.01
Chewing difficulty	Yes, *n* (%) ^a^	3831 (12.7)	135 (19.5)	<0.001

^a^ *p* value was calculated using the chi-squared test. ^b^ *p* value was calculated using the Mann-Whitney U test. Abbreviations: BMI, body mass index.

**Table 3:** Crude odds ratios and 95% CI for high HbA1c levels according to the analyzed factors in all participants.

Variables		Crude ORs	95% CI	*p* Value
Gender	Women	1	(reference)	<0.001
	Men	2.457	2.076–2.908	
Age (years)		1.066	1.058–1.075	<0.001
BMI (kg/m^2^)		1.235	1.215–1.254	<0.001
Current smoker	No	1	(reference)	<0.001
	Yes	1.802	1.524–2.131	
Regular exercise habits	Presence	1	(reference)	<0.05
	Absence	0.819	0.691–0.970	
Physical activity	High	1	(reference)	0.598
	Low	1.048	0.881–1.245	
Sleeping well	Yes	1	(reference)	0.333
	No	1.079	0.925–1.259	
Alcohol drinking	Not everyday	1	(reference)	0.933
	Everyday	0.993	0.834–1.181	
Eating speed	Not quickly	1	(reference)	<0.01
	Quickly	1.279	1.098–1.489	
Chewing difficulty	No	1	(reference)	<0.001
	Yes	1.671	1.381–2.023	

Abbreviations: ORs, odds ratios; CI, confidence interval; BMI, body mass index.

**Table 4:** Adjusted odds ratios and 95% CI for high HbA1c levels according to the analyzed factors in all participants.

Variables		Adjusted ORs	95% CI	*p* Value
Model 1				
Gender	Women	1	(reference)	<0.001
	Men	2.120	1.777–2.529	
Age (years)		1.070	1.061–1.079	<0.001
Current smoker	No	1	(reference)	<0.001
	Yes	1.607	1.345–1.921	
Eating speed	Not quickly	1	(reference)	<0.01
	Quickly	1.302	1.115–1.520	
Chewing difficulty	No	1	(reference)	<0.01
	Yes	1.297	1.067–1.576	
Model 2				
Gender	Women	1	(reference)	<0.001
	Men	1.568	1.310–1.878	
Age (years)		1.077	1.068–1.087	<0.001
BMI (kg/m^2^)		1.246	1.225–1.268	<0.001
Current smoker	No	1	(reference)	<0.001
	Yes	1.566	1.303–1.882	
Chewing difficulty	No	1	(reference)	<0.05
	Yes	1.302	1.065–1.591	

Abbreviations: ORs, odds ratios; CI, confidence interval; BMI, body mass index. Model 1: Adjusting gender, age, smoking status, exercise habits, eating speed and chewing status. Model 2: Adjusting gender, age, BMI, smoking status, exercise habits, eating speed and chewing status.

## Data Availability

The data presented in this study are available on request from the corresponding author. The data are not publicly available due to privacy and ethical constraints of the subject.
